# Prevalence of Congenital Coronary Artery Anomalies and Variants in 2697 Consecutive Patients Using 64-Detector Row Coronary CTAngiography

**DOI:** 10.5812/iranjradiol.8070

**Published:** 2012-09-17

**Authors:** Abbas Arjmand Shabestari, Shahram Akhlaghpoor, Reza Tayebivaljozi, Farzaneh Fattahi Masrour

**Affiliations:** 1 Cardiac CT Department, Modarres Hospital, Shahid Beheshti University of Medical Sciences, Tehran, Iran; 2 CT Department, Noor Medical Imaging Center, Tehran, Iran; 3 Advanced Diagnostic and Interventional Radiology Research Center (ADIR), Tehran University of Medical Sciences, Tehran, Iran; 4 Radiology Department, Sina Hospital, Tehran University of Medical Sciences, Tehran, Iran

**Keywords:** Coronary Artery Disease, Tomography,X-Ray Computed, Patients

## Abstract

**Background:**

Coronary artery anomalies are not common, but could be very serious.

**Objectives:**

This study determines the frequency of coronary anomalies and normal variants by multi-detector-row computed tomography (MDCT).

**Patients and Methods:**

The results of cardiac MDCT study in 2697 consecutive patients were analyzed retrospectively. Acquisition was performed by a 64-detector row CT machine. Imaging results were assessed by experienced radiologists.

**Results:**

Myocardial bridging was by far the most frequent coronary variant (n = 576, 21.3%). Eighty-three subjects (3.1%) showed other coronary anomalies and variants. Anomalies of origination and course of the left main coronary artery (LMCA) were detected in 1.09% of the subjects. The frequency of these anomalies in the right coronary artery (RCA), left circumflex artery (LCx), left anterior descending artery (LAD), posterior descending artery (PDA) and obtuse marginal (OM) artery were 1.24%, 0.33%, 0.1%, 0.07% and 0.03%, respectively. The single coronary pattern was seen in 0.18% and coronary fistulas in 0.07%.

**Conclusion:**

Based on the fact that coronary CT-angiography using MDCT can display different coronary anomalies, this study shows similar results to other reports on the subject. Future advances in the performance of CT machines will further improve the quality of CT-based cardiac imaging.

## 1. Background

Coronary artery anomalies are a vast and diverse group of congenital anomalies with different symptoms and pathophysiologic mechanisms (1). Most physicians are familiar with them because of their potential to induce sudden death, especially in young athletes (2-5). It is estimated that the prevalence of congenital coronary anomalies is 1-2% in the general population, but figures between 0.3 to 5.6% have been mentioned in the literature (6-14). Currently, no generally accepted classification exists and the definition of normality and abnormality has not yet been completely clarified (2).Some researchers classify coronary anomalies based on their hemodynamic significance, while others believe in exclusively anatomic classification (2, 3, 8). Coronary anomalies can present with chest pain, dyspnea, arrhythmia, syncope and other symptoms. Some of them are just benign anatomic variants, while others are considered to be significant and even malignant (2, 7, 15). Typical examples of malignant anomalies are anomalous origination of the coronary artery from the opposite Valsalva sinus of the aorta and its interarterial course between the aorta accompanied by the pulmonary artery, which may cause syncope or even sudden death and anomalous origination of the left coronary artery from the pulmonary artery (ALCAPA), which can result in resting angina (2). Fifty-five to 93% of patients who die because of malignant coronary anomalies have no previous alarming sign and only 10% of them undergo coronary study before death (2, 16-18). In some studies, up to 33% of sudden cardiac deaths in the youth have been related to these malignant anomalies and the prospective risk of mortality among individuals younger than 40, who have an abnormal origination of the coronary artery from the opposite sinus with an interarterial course is estimated to be as high as 30% (7, 8, 11, 12, 16, 19). On the other hand, even benign anomalies can have clinical significance; for example, in high take off site of coronary arteries, clamping of the aorta during surgery may cause cardiac ischemia (8). In recent years, advances in software and hardware of multi-detector row computed tomography (MDCT) machines have probably made them the best non-invasive modality for diagnosing congenital coronary artery anomalies, since MDCT can show up to 95% of all coronary artery segments (6, 7, 20, 21).

## 2. Objectives

Over the past years, different types of rare coronary artery anomalies have been reported (22, 23), but to the best of our knowledge, no large scale study of coronary anomalies has been performed using 64-detector row CT. The aim of this study was the retrospective review of coronary anomalies and variants in a group of consecutive patients scanned in current practice and to determine its frequency.

## 3. Patients and Methods

### 3.1. Patients

More than 2750 coronary CT scans were performed in our center from March 2005 to March 2010. The imaging results of 2697 of these patients, consisting of 1027 females (38%) and 1670 males (62%), were satisfying enough to enter the study. A small number of cases (n = 58) were dropped because of the poor quality of the images. A retrospective review of these imaging results was performed to identify the patients with coronary anomalies and normal variants. The mean age of the patients was 55 years (range, 10 to 98 years). Most of them were referred for evaluation of coronary artery disease (84%, n = 2265). Other indications (16%, n = 432) mainly included suspected coronary anomaly, inconclusive conventional coronary angiography, determining precise 3-dimentional path of the coronary arteries and searching for the possible cause of the patient’s cardiac symptoms. The post-coronary artery bypass graft (post-CABG) and post-stenting patients were included in this study. Prior to performing CT, all patients had signed an informed consent stating that their imaging results may be used anonymously for research purposes. Local ethics in research committee of our center had approved this study.

### 3.2. Imaging Data Acquisition and Reconstruction Protocol

Using a 64-detector row CT scanner (SOMATOM Sensation 64, Siemens Medical Solutions; Forchheim; Germany), coronary CT angiography with synchronous electrocardiographic tracing of the patients was performed. The scanning technique and its parameters have previously been addressed and pointed out elsewhere (24). In summary; approximately 80 to 100 mL of non-ionic contrast medium was injected at a rate of 4-5 mL/s, followed by 40-50 mL of saline solution chaser through a dual-head injector. Applied MDCT-angiography parameters were as follows: collimation width 64 × 0.6 mm, tube potential 120 kV, tube effective current 650 to 850 mA, tube rotation time 330 ms, table feed 3.8 mm/rotation, temporal resolution 83 to 165 ms, scanning time 8 to 13 seconds in an inspiratory breath hold and scan field from tracheal carina to the diaphragm.The axial, coronal, sagittal and oblique multiplanar reconstruction; thin-slab maximum intensity projection; and volume-rendered images were reformatted on either an on-line workstation (Wizard, Siemens Medical Solutions; Erlangen; Germany) or an offline one (MMWP; Siemens Medical Solutions; Erlangen; Germany). Multiplanar reconstructed views were reformatted using a thickness of 0.6 mm with 0.4-mm intervals and the image thickness in maximum intensity projection reconstructions was either 5 or 6 mm. Different retrospective electrocardiography-gated reconstruction temporal window settings, usually between 30% to 75% of the consecutive R waves (R-R interval) in gated ECG were applied and most reconstructions were best achieved in mid- to end-diastole (from 60% to 70% of R-R intervals).

### 3.3. Interpretation of Results

Interpretation of MDCT-angiography images of the patients was made independently by two radiologists who had at least three years of experience in cardiac CT. In case of disagreement on interpretation, the radiologists would discuss the case with a third radiologist to reach a consensus. The origin and course of coronary arteries, their branches and territories, coronary calcium score and presence and severity of plaques and stenoses were retrospectively determined in all patients. The criteria used for normality in our institute correlated nearly to that of Angelini’s “Normal Features of the Coronary Anatomy in Humans” (1). We assumed that normally there are two coronary ostia which are located in the right and left anterior sinuses (upper mid-section); there is only one normal common trunk on the left; proximal orientation of a coronary artery should be between 45 to 90 degrees off the aortic wall; a normal coronary artery should travel directly from the ostium to destination and has a subepicardial (extramural) course; it should have adequate branches for the dependent myocardium and should terminate in the capillary bed. Essential territories were the right ventricular free wall, antero-septal and left ventricular free wall. We did not intend to define what a normal variant is and what an abnormality is; rather, we wanted to report even the smallest breaches of accepted normal coronary anatomy which may alter a surgeon’s approach or a patient’s outcome. Myocardial bridging is also in contravention of the normal subepicardial course of coronary arteries which can potentially be important, so we included cases of myocardial bridging in our series as well. We divided myocardial bridging into two types; superficial, with a thin covering band or layer (less than 1 mm thick) and no deviation of the vessel into the myocardium; and deep, where the vessel moves well down into the myocardium (more than 1 mm thickness of the covering myocardial layer).

## 4. Results

A comprehensive classification of coronary artery anomalies has been described by Angelini et al.(3). However, for practical purposes, we used a simplified categorization. Twenty three percent of our patients (n = 619) had some kind of coronary anomaly or normal variant, including myocardial bridging (Table 1). The age range of these patients was 10 to 84 years. Myocardial bridging was by far the most frequent normal variant. Four individuals (0.1%) out of all enrolled subjects were referred for assessment of suspected coronary anomalies diagnosed in their catheter coronary angiographies. CT-angiography appropriately showed anomalous coronary arteries in all of them and their three-dimensional relationship with other contiguous structures were better shown when compared with catheter angiography results.

**Table 1:** Prevalence of Coronary Artery Anomalies and Normal Variants

**Coronary Anomalies/Variants**	**Percent (No)**
**Myocardial Bridging**	21.3 (576)
Superficial	14.7 (399)
Deep	6.6 (180)
**Anomalies of LMCA ^a^**	1.1 (30)
Absent LMCA	0.7 (19)
Origination from RSV^a^	0.07 (2)
Acute angulation at origin	0.22 (6)
Other	0.11 (3)
**Anomalies of RCA ^a^**	1.25 (34)
Origination from LSV or NSV ^a^	0.48 (13)
Acute angulation at origin	0.44 (12)
Other	0.33 (9)
**Anomalies of LCX ^a^**	0.33 (9)
Absent LCx	0.07 (2)
Origination from RSV	0.26 (7)
**Single Coronary Pattern**	0.18 (5)
**Fistulas**	0.07 (2)
**Other**	0.22 (6)

^a^Abbreviations: LCx; left circumflex artery, LMCA; left main coronary artery, LSV; left sinus of Valsalva, NSV; non-coronary sinus of Valsalva, RCA; right coronary artery, RSV; right sinus of Valsalva

### 4.1. Myocardial Bridging

Myocardial bridging was found in 21.3% of our patients (n = 576). Sixty five percent of these patients were male (n = 375) and 35% were female (n = 201). In 99.5% of the subjects with bridging (n = 573), there was only one tunneled artery, consisting of the left anterior descending (LAD) artery in 98% (n = 565), the right coronary artery (RCA) in 1.2% (n = 7) and the left main coronary artery (LMCA) in 0.1% (n = 1) of cases. There were three patients (0.5%) who had simultaneous bridging in two arteries: both LAD artery and RCA had tunneled segments in these three patients. Interestingly, in the single case of LMCA bridging, the vessel had commenced from the right Valsalva sinus and taken a path through the interventricular septum. In two out of seven subjects with RCA bridging, these vessels had an abnormal site and angulation while arising from the right Valsalva sinus. Sixty nine percent of the bridged segments were of superficial type (n = 399). The remaining 31% (n = 180) had deep bridging, all occurring in LAD.

### 4.2. Anomalies of LMCA

**Figure 1:**
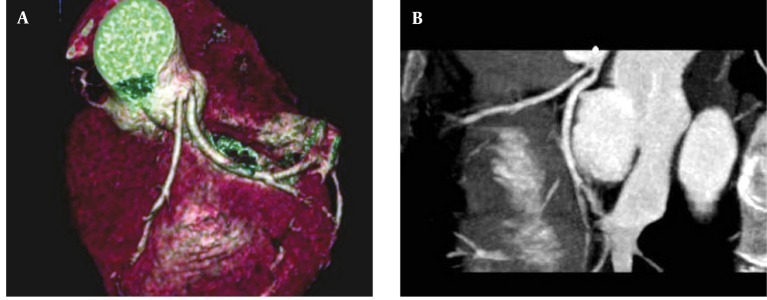
(A and B) Absence of the left main coronary artery with separate ostia of the left anterior descending artery and left circumflex artery from the left Valsalva sinus in a 52-year-old man A, Volume rendering reconstruction; B, Maximum intensity projection

**Figure 2:**
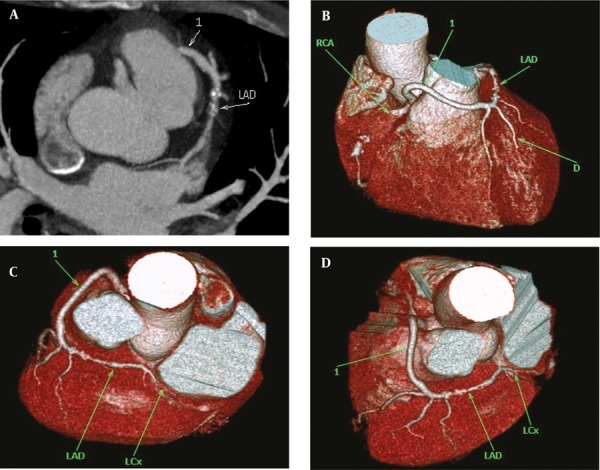
(A, B, C and D) Maximum intensity projection and volume rendering reconstructions of a case of absent left main coronary artery, a large anomalous arterial structure (1) with pre-infundibular course supplies both LADs, LCx and their branches. RCA is occluded. D, Diagonal artery Abbreviations: LAD, Left Anterior Descending; LCx, Left Circumflex; RCA, Right Coronary Artery

In 19 patients (0.7%) LMCA was absent, out of which 18 (0.66%) had separate ostia for LAD and left circumflex (LCx) arteries (Figure 1). In the last case with absent LMCA (0.03%), a large anomalous arterial structure with a pre-infundibular course, which had arisen from the aortic right Valsalva sinus, supplied both LAD and LCx arteries (Figure 2).

**Figure 3:**
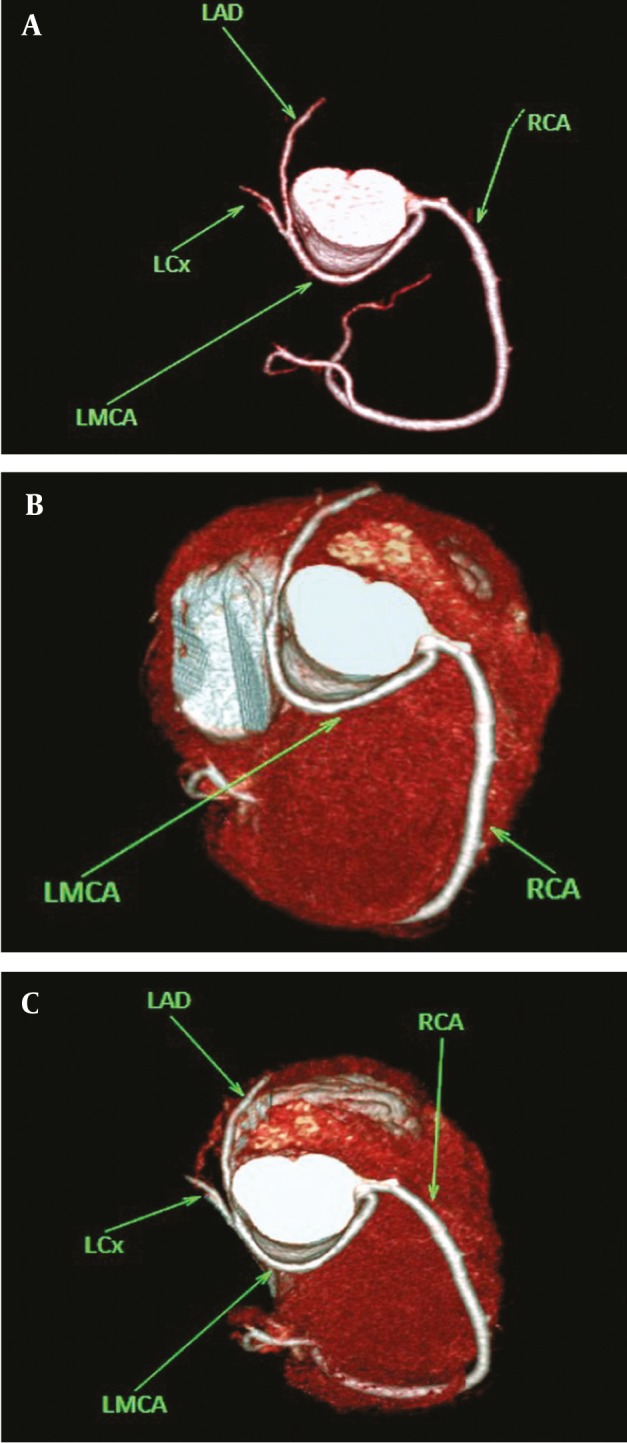
(A, B and C) Aberrant commencement of LMCA from right sinus of Valsalva with retro-aortic course in a 46-year-old man Abbreviations: LAD Left Anterior Descending; LCx, Left Circumflex; LMCA, Left Main Coronary Artery; RCA, Right Coronary Artery

**Figure 4:**
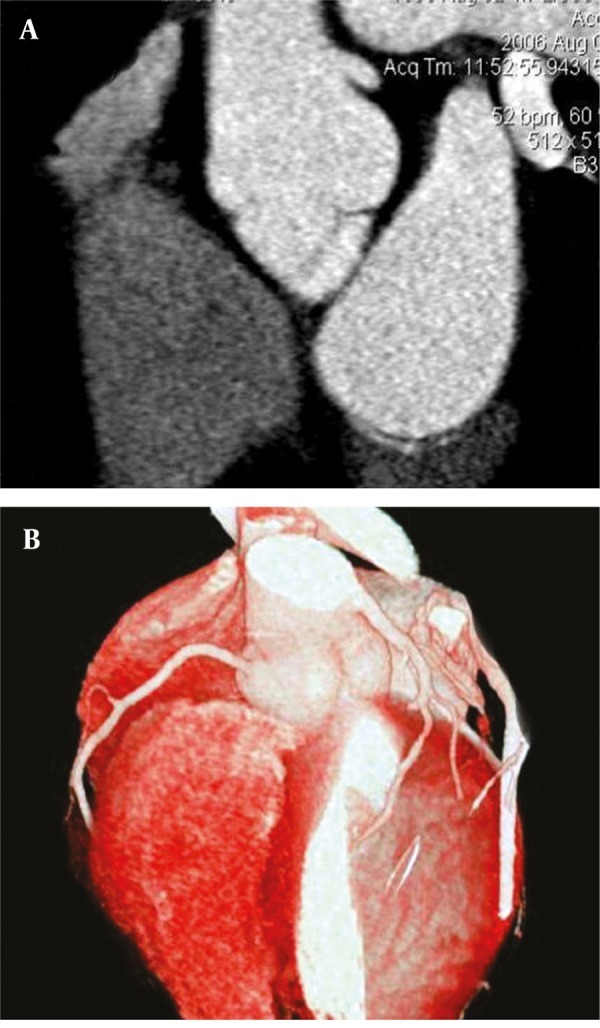
(A and B) A 49-year-old woman with high take-off site of left main coronary artery from aorta A, Maximum intensity projection and B, Volume rendering reconstructions

Abnormal origination of LMCA from the right Valsalva sinus was depicted in two patients (0.07%); nevertheless, none of them had an interarterial course (Figure 3). In one case (0.03%), LMCA originated from the posterior part of the ascending aorta (Figure 4). Acute and sharp angulation of LMCA origin was detected in six (0.22%) subjects, out of which three had concurrent acute-angle origination of RCA. Two patients (0.07%) had LMCA with abnormal location in the left Valsalva sinus; one in the far superior part of the sinus and the other in its far lateral part.

### 4.3. Anomalies of RCA

**Figure 5:**
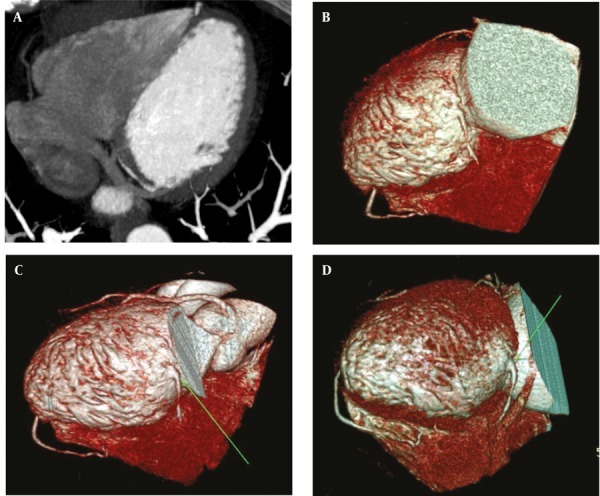
(A, B, C and D) Maximum intensity projection and volume rendering reconstructions demonstrating origination of an anomalous artery (arrow) from left ventricle in a 56-year-old woman The artery reaches the posterior interventricular groove. There is a small tunneled segment in this artery causing smooth narrowing. RCA is rudimentary. Abbreviation: RCA, Right Coronary Artery

**Figure 6:**
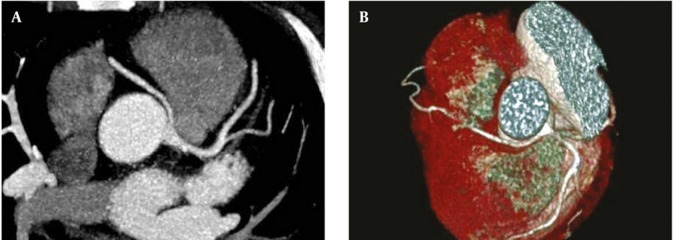
(A and B) Anomalous origination of the right coronary artery from the left sinus of Valsalva with interarterial course in a 52-year-old woman

There was a single case (0.03%) of RCA agenesis, SA-nodal and the right ventricular arteries revealing direct and separate origins from the right Valsalva sinus. Another single subject (0.03%) was found whose RCA was diminutive and had an anomalous small arterial structure directly arising from the posterior aspect of the left ventricle, traversing the myocardium and entering the epicardial fat space to reach the posterior interventricular groove (Figure 5). Twelve cases (0.44%) of anomalous origination of RCA from the left sinus of Valsalva were seen; all but one had a malignant (interarterial) course between the aorta and the pulmonary artery (Figure 6). In one patient (0.03%), RCA had arisen from the non-coronary sinus. Twelve subjects (0.44%) had acute angulation of RCA origin, three of which as mentioned before had simultaneous acute angulation of LMCA and RCA origins. In seven patients (0.26%), the RCA ostium had an abnormal location in the right Valsalva sinus; consisting of two high take off sites, one low, one lateral and three medial positions of ostium. In three cases with the medial location of RCA ostium in the right sinus of Valsalva, the vessel had a partial inter-arterial course.

### 4.4. Anomalies of LCx Artery

**Figure 7:**
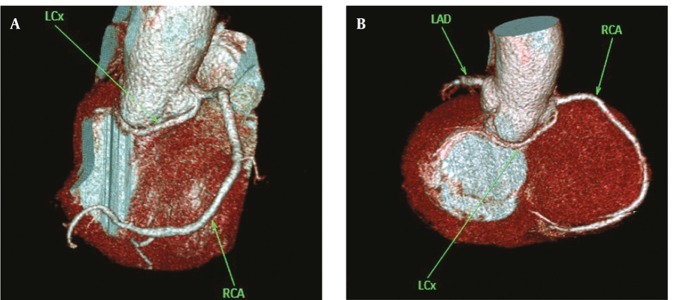
(A and B) Anomalous origination of LCx from right sinus of Valsalva with retro-aortic course in two patients A, 53 year-old woman; B, 64 yearold man a B Abbreviations: LAD, Left Anterior Descending; LCX, Left Circumflex; RCA, Right Coronary Artery

LCx artery was absent in two patients (0.07%). LCx ostium was located in the right sinus of Valsalva in seven cases (0.26%), all showing a postero-inferior course to the aorta (retro-aortic path) (Figure 7).

### 4.5. Anomalies of LAD Artery

**Figure 8:**
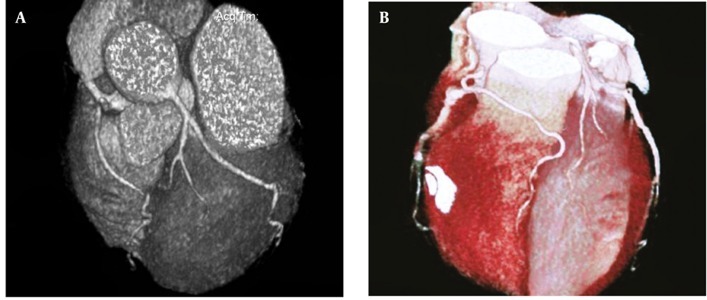
(A and B) Maximum intensity projection and volume rendering reconstruction show rudimentary LAD artery in a 49-year-old man Its distal territory is supplied by an artery which has arisen from right coronary artery and has a pre-infundibular course Abbreviation: LAD, Left Anterior Descending

In one case (0.03%), there was a rudimentary LAD artery, the distal territory supply being derived from an artery which had arisen from RCA with a course anterior to the pulmonary artery (pre-infundibular course) (Figure 8). Diagonal continuation of LAD artery was observed in two patients (0.07%).

### 4.6. Anomalies of Other Coronary Arteries

There was one case (0.03%) of direct commencement of the posterior descending artery (PDA) from the right Valsalva sinus. One case of absent posterior descending artery (PDA) (0.03%) and another single case of absent obtuse marginal (OM) artery (0.03%) were also seen.

### 4.7. Single Coronary Pattern

Five cases (0.18%) of single coronary ostium were found. In all of them, the ostium was located in the right Valsalva sinus and the LMCA, arising from RCA, had an interarterial course.

### 4.8. Fistulas

**Figure 9:**
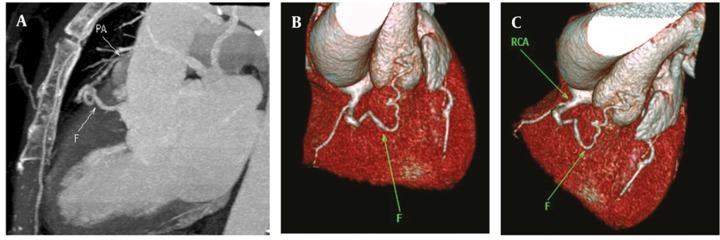
(A, B and C) Maximum intensity projection and volume rendering reconstructions showing fistulous connection (F) between RCA and pulmonary artery (PA) in a 50-year-old woman

We identified two cases (0.07%) of coronary-to-pulmonary artery fistulas. In one of them, bilateral coronary-topulmonary artery fistulous connections were observed involving RCA on one side and LMCA-LAD artery on the other side. The other patient had RCA to main pulmonary artery fistula (Figure 9).

## 5. Discussion

Myocardial bridging is defined as an intra-myocardial segment in the course of a major epicardial coronary artery (25, 26). The most common site of bridging is in the middle segment of the LAD artery (25). It is reported that myocardial bridging may have caused ischemia and acute coronary syndrome, coronary spasm, rupture of ventricular septum, arrhythmia and sudden death (27). Some researchers divide LAD artery bridging into two or three subtypes, according to the depth and course of the tunneled segment (12, 26, 28, 29). However, it is suggested that even a thin layer of muscle or fibrous-fatty tissue may cause compression of coronary arteries in systole (28). Prevalence of myocardial bridging has been reported to be varying from 0.5 to 16% in angiographic series and from 40 to 80% in autopsy studies (30). This difference in prevalence implies a relatively low diagnostic sensitivity of conventional angiography in which demonstration of tunneled segments depends on many factors (31). MDCT is an alternative and non-invasive method for demonstration of myocardial bridging and potentially can even show the rare right ventricular intracavitary course of LAD artery (28). In our study, like similar studies, the frequency of myocardial bridging was higher than that determined by angiographic series (26, 28). This higher frequency could suggest a higher diagnostic sensitivity of MDCT in this regard, when compared to catheter angiography, though this study was not designed to compare the two modalities. However, hemodynamic significance of tunneled segment, when reconstructions are limited to only diastolic phases of cardiac cycle, may not be determined by MDCT, since it may not allow demonstration of the classic milking sign during systole (28). In our study, the frequency of coronary artery anomalies and variants other than myocardial bridging was 3.1% (n = 83) which is higher than the range of 0.3% to 2.2% found in conventional angiographic and autopsy series (7, 15, 30, 32). However, by changing the criteria of normalcy and excluding the referral bias, these figures may change which may in part explain the inconsistencies between the results of different studies.

Absence of LMCA, suggested to be a normal variant rather than an anomaly, has little clinical significance, but recognizing this condition before coronary bypass surgery can be helpful (1, 2). Its frequency was 0.66% in our study. Most cases of abnormal origination from the opposite sinus occurred in RCA (n = 12); all but one having an interarterial course. Illustration of this anomaly occurs more frequently in cardiac CT and MR than in conventional angiography (7). The interarterial path of RCA has been linked to various cardiac symptoms, ranging from exertional angina to myocardial infarction (7). The LMCA origination from the right sinus of Valsalva was observed in two cases, neither with an interarterial course; nonetheless, this anomaly can also be associated with cardiac symptoms (7). No patient with the origination of the left coronary artery from the pulmonary artery was found in our study; this anomaly is a serious one and its rate of mortality in infancy can be as high as 90%. Therefore, it is not surprising that we could not find such a case in our adult population (32). While several researchers have suggested that the incidence of anomalous origination of LCx artery could be as high as 0.67%, its frequency in our study was 0.26% (n = 7) and in all cases, the LCx artery course was between the aorta and the left atrium. This aberrant path can potentially complicate aortic valve surgery (7, 9, 12). Single coronary pattern is a rare condition, for which the prevalence values of 0.0024% to 0.044% have been mentioned (33). The coronary tree may have different patterns in each individual case of single coronary ostium. This condition may cause severe cardiac ischemia in coronary atherosclerotic disease and if associated with an interarterial course of a major coronary branch can result in an increased risk of sudden death (33). The frequency of single coronary ostium in our study population was 0.18% (n = 5) which is remarkably higher than the above-mentioned figures. All of our five cases had an interarterial path. Coronary artery fistulas are reported in 0.1-0.2% of cases undergoing conventional coronary angiography (33). When a fistulous coronary artery terminates into a right-sided cavity, it causes steal phenomenon and can potentially lead to insufficient cardiac perfusion (2, 33). We encountered two cases (0.07%) of fistulas (which were assumed to be congenital), both of which drained into the main pulmonary artery. However, the most common site of drainage reported in the literature has been the right ventricle (33).

Demonstration of an anomalous or aberrant coronary vessel prior to operation or intervention is extremely crucial (29, 32, 34). The majority of congenital coronary anomalies are asymptomatic and considering the recent increase in the number of interventional procedures, their importance has become clearer (34). The aberrant artery may be transected or excluded from the circulation during cardiac surgery. It may prolong a cardiac operation/intervention or make it ineffective. Abnormal location of the coronary ostia can complicate aortic valve surgery (32). Many angiographers may not be experienced enough to catheterize every anomalous coronary vessel. In such conditions, inability to identify the coronary ostia in their normal anatomic locations brings about the diagnosis of coronary anomaly in the patient (32, 34). Even in experienced hands, the procedure of catheterization of such vessels is cumbersome and time-consuming; moreover, it needs additional projections (32).Cardiac MDCT can prove invaluable in these occasions. It has been shown that in a group of 35 patients with coronary anomalies (diagnosed by conventional angiography), MDCT was able to depict 100% of the anomalies (29). By applying dose reduction algorithms, the radiation dose of a cardiac CT can be reduced to that of a coronary arteriography (approximately 5 millisieverts) or even less (6, 35-37). In addition, MDCT is much less invasive and less operator-dependent than catheter angiography (6). Catheter angiography which has been regarded as the gold standard is expensive and invasive and may sometimes fail to completely show the complex nature of these anomalies (6, 32, 38, 39). In a study conducted by Shi et al., conventional angiography was unable to demonstrate 47% of the coronary anomalies which had been diagnosed by MDCT (30). In two other studies, conventional arteriography could show only 50-55% of the anomalies seen by MDCT (7, 32). On the other hand, other modalities have their own limitations. Poor acoustic window and the patient’s body structure may hinder echocardiographic visualization of the coronary arteries (6, 8). Currently, cardiac MRI has lower spatial resolution than MDCT and is more prone to cardiac and respiratory motion-related degradations (6, 8, 29). In our experience, cardiac CT is an elegant and noninvasive tool for assessment of coronary arteries with a high degree of anatomic accuracy; in most cases, it can achieve adequate visualization of the coronaries. Regarding study limitations some points should be considered. Our institute is a cardiac CT referral center; so, like other studies of this kind, our study suffers from referral bias, so that the frequency of coronary anomalies and normal variants may be overestimated and cannot be regarded as the confidently true frequency of the entity in the general population. On the other hand, since we usually do not candidate patients with tachy-arrhythmia for cardiac CT, most of our patients had normal heart rate and rhythm; thus, we were unable to assess tachy-arrhythmic patients, which may lead to a reduced estimation of true frequency of coronary anomalies.

Cardiac CT-angiography using MDCT may depict different coronary anomalies and normal variants. It is less expensive and less invasive than catheter-based arteriography and can potentially have comparable or even better results. Its three-dimensional capabilities can display the anatomy of coronary arteries conspicuously. In the future, with improvements in temporal and spatial resolution and radiation dose reduction, MDCT can overcome its current limitations, further increasing its role in coronary artery assessments.
